# *Oenococcus oeni* Exopolysaccharide Biosynthesis, a Tool to Improve Malolactic Starter Performance

**DOI:** 10.3389/fmicb.2018.01276

**Published:** 2018-06-12

**Authors:** Maria Dimopoulou, Jerôme Raffenne, Olivier Claisse, Cécile Miot-Sertier, Nerea Iturmendi, Virginie Moine, Joana Coulon, Marguerite Dols-Lafargue

**Affiliations:** ^1^Université de Bordeaux, EA 4577 Œnologie, INRA, USC 1366, ISVV, Bordeaux INP, Villenave-d'Ornon, France; ^2^Biolaffort, Bordeaux, France

**Keywords:** *Oenococcus oeni*, malolactic fermentation, exopolysaccharides, stress, wine

## Abstract

*Oenococcus oeni* is the lactic acid bacterium that most commonly drives malolactic fermentation (MLF) in wine. Though the importance of MLF in terms of wine microbial stability and sensory improvement is well established, it remains a winemaking step not so easy to control. *O. oeni* displays many adaptation tools to resist the harsh wine conditions which explain its natural dominance at this stage of winemaking. Previous findings showed that capsular polysaccharides and endogenous produced dextran increased the survival rate and the conservation time of malolactic starters. In this paper, we showed that exopolysaccharides specific production rates were increased in the presence of single stressors relevant to wine (pH, ethanol). The transcription of the associated genes was investigated in distinct *O. oeni* strains. The conditions in which eps genes and EPS synthesis were most stimulated were then evaluated for the production of freeze dried malolactic starters, for acclimation procedures and for MLF efficiency. Sensory analysis tests on the resulting wines were finally performed.

## Introduction

*Oenococcus oeni* is the bacterial species most frequently responsible for malolactic fermentation (MLF) in wine (Lonvaud-Funel, 1999). MLF is an important qualitative winemaking step, taking place after the alcoholic fermentation, and whose main consequence is the conversion of L-malic acid to L-lactic acid. It can be driven by the autochthonous *O. oeni* strains, or by commercialized selected strains (starters), massively introduced to wine usually at the end of the alcoholic fermentation (Davis et al., 1985). *O. oeni* ability to be naturally selected among great diversity of microorganisms in wine and to drive MLF in such hostile environment is not fully understood even although many mechanisms have been described.

Most *O. oeni* strains are acidophilic, able to grow at pH 3.5 or lower, tolerant to the presence of ethanol, sulfur dioxide and polyphenols as well as resistant to low temperature (Lonvaud-Funel, 1999). This high level of tolerance to wine and winemaking is linked to metabolic equipment dedicated to stress tolerance (Guzzo et al., 1997; Delmas et al., 2001; Grandvalet et al., 2005; Beltramo et al., 2006; Favier et al., 2012; Maitre et al., 2014), to resistance to low pH (Ramos et al., 1995; Tourdot-Maréchal et al., 1999; Martin et al., 2005; Olguín et al., 2009), or ethanol (Garbay and Lonvaud-Funel, 1996; Da Silveira et al., 2003; Chu-Ky et al., 2005; Olguín et al., 2015; Mendoza et al., 2017; Bonomo et al., 2018; Contreras et al., 2018). Some of the mechanisms explaining *O. oeni* tolerance to wine are strain specific, like those implicated in rare nutrient transport and metabolism (Garvie, 1967; Dicks et al., 1995; Borneman et al., 2010; Jamal et al., 2013), in biogenic amine production (Lucas et al., 2008; Cañas et al., 2009; Costantini et al., 2009; Pramateftaki et al., 2012) or in resistance to bacteriophages (Poblet-Icart et al., 1998; São-José et al., 2004; Jaomanjaka et al., 2013), while others are shared by all the strains in the species (Bartowsky and Borneman, 2011; Borneman et al., 2012; Betteridge et al., 2015).

Exopolysaccharides (EPS) production can also contribute to *O. oeni* protection. EPS are macromolecules constituted of repeated units of sugar molecules and excreted by the bacteria. In lactic acid bacteria, they can accumulate around the cell and form a thin to thick capsule depending on the strain and/or they can be liberated in the surrounding medium (Cerning, 1990; De Vuyst and Degeest, 1999; Degeest, 2001; Ruas-Madiedo and de los Reyes-Gavilán, 2005). Indeed 75% of the *O. oeni* strains examined are capsulated with exopolymers made of glucose, galactose and rhamnose, and also liberate exopolymers in various proportions depending on the strain considered (Ciezack et al., 2010; Dimopoulou et al., 2012, 2014). According to genotype/phenotype correlations, the genes dedicated to capsule synthesis are located in a cluster called *eps2*, highly variable in gene composition (Dimopoulou et al., 2012, 2014, 2016). Part of the EPS capsule may be liberated along the culture and some strains also produce EPS in a soluble form (Ciezack et al., 2010; Dimopoulou et al., 2014). Half of the strains examined also produce dextran in the presence of sucrose, thanks to the presence of *dsrO* gene (Dimopoulou et al., 2014). The *O. oeni* strain tolerance to freeze-drying is highly improved when the strains are capsulated and produce dextran. EPS may also help the cell to overcome the stress linked to inoculation in wine (Dimopoulou et al., 2016). However, recent transcriptomic studies did not highlight any significant regulation of *eps* genes in a context of wine synthetic media with or without stress (Olguín et al., 2015; Bastard et al., 2016; Margalef-Català et al., 2016; Liu et al., 2017).

In order to better understand the role of EPS in *O. oeni* adaptation to wine, we examined both EPS production and *eps* gene expression in several strains able to produce such polymers and representative of the species diversity. The *eps* gene expression was analyzed along growth and in the presence of different stresses, as well as in wine. This highlighted the conditions possibly stimulating EPS formation. Based on these results, we then examine production and inoculation methods leading to better malolactic starter survival, and verify that these methods preserve wine quality.

## Materials and methods

### Microbial strains

The list of strains studied and their origin are indicated in Table 1.

**Table 1:** Specific production of EPS (mg/l/AU^600^) of several *O. oeni* strains, selected for describing the *O. oeni* species genetic diversity and EPS producing abilities according to Dimopoulou et al. (2014).

**Name^a^**	**Collection**	**Origin**	**EPS/Absorbance (mg/l/UA)**					
			**solid SMD, pH4.8**	**SMD pH 4.8**	**SMD pH4**	**SMD pH 3.5**	**SMD 4.8 + ethanol 5%**	**SMD 4.8 + sucrose^b^**
VF	Commercial	Starter VF, Martin Vialatte	2	0	0	4	5	34
L40_4	IOEB	Red wine Lebanon	2	1	0	3	12	341
B419	AWRI	Lalvin EQ54 Lallemand	4	3	8	8	12	51
S19	S	Red wine France	3	6	3	5	12	25
436a	IOEB-S	Red wine Bordeaux France	6	8	8	11	11	2345
1491	IOEB	Red wine France	5	9	12	14	16	238
B10	IOEB	French isolate	1	2	3	11	16	21
O608	IOEB	French isolate	3	8	7	12	14	12
Ci Ne	IOEB	Starter CHR Hans en	5	12	13	21	25	34
L26_1	IOEB	Leba nonisol ate	2	12	14	17	43	42 ^T^
S11 B429	SAWRI	Sparkling white wine France	12	15	14	21	32	2431
		Lalvin VP41 Lallemand	4	16	17	19	43	310
S25	S	Red wine France	5	17	15	27	43	32 ^T^
S28	S commercial	B28 PreAc, Laffort, France	4	19	18	25	34	89
C52	IOEB	Cider Normandy France	3	21	23	26	36	36 ^T^
O607	IOEB	Frenchisolate	3	24	26	27	22	32 ^T^
L65_2	IOEB	Redwine, Lebanon	3	28	32	47	57	34
S22	S	Sparklingwhite wine Bourgogne France	6	29	18	39	49	21 ^T^
S13	S	Redwine France	10	30	27	45	67	32
S23	S	white wine, England	12	31	32	41	88	421
L18_3	IOEB	Red wine Lebanon	6	32	34	45	67	34
S161	S, commercial	350 PreAc, Laffort France	7	33	34	45	54	42
9304	IOEB	Ci der France	14	34	32	47	321	876
9803	IOEB	Red wine France	25	39	58	69	132	2439
ATCC BAA-1163	ATCC	Red wine, France	16	43	46	65	87	1342
C23	IOEB	Ci der Normandy France	12	43	41	57	89	42
8417	IOEB	Ropy red wine, France	21	45	43	86	78	987
C28	IOEB	Cider, Bretagne France	19	46	45	76	112	89
PSU-1	commercial	Red wine USA	20	53	57	76	143	100
9805	IOEB	Red wine France	21	54	56	75	123	2578
S14	S	Red wine France	4	65	54	88	94	62
450	IOEB-S commercial	450 PreAc,Laffort, France	13	67	69	83	112	54
S12	S	White wine France	13	67	78	87	122	332
B418	AWRI	MCW Lallemand	21	69	74	98	99	236
O501	IOEB	Red wine France	12	75	85	124	234	1285
9517	IOEB	Flocde Gas cogne France	17	87	98	99	123	82
B548	AWRI	BL-01 Lallemand	7	98	112	132	200	87
O205	IOEB	Champagne isolate	14	109	132	165	177	96
B16	IOEB commercial	B16, Laffort France	34	112	114	198	342	117
B422	AWRI	Viniflora CHR35, Chr. Hanen	24	118	96	132	175	104
S15	S	Red wine France	8	123	143	165	245	100
O502	IOEB	French isolate	23	153	165	187	321	1789
277	IOEB-S commercial	SB3, Laffort France	12	154	167	199	248	1332
Mean^c^			12^*^	54	57	75 ^*^	115 ^*^	550 ^*^
SD			8	41	45	54	90	794

*The strains were grown for 15 days in the indicated culture conditions before EPS and biomass quantification*.

a *The strains whose EPS production is underlined in gray do not display any eps2 gene cluster according to Dimopoulou et al. (2012, 2014) and the corresponding results were not use for calculating the means. The strains whose name is underlined in color represent 3 distinct families of strains further examined in this paper*.

bT *: Strains displaying a truncated dsrO gene encoding a non-functional dextransucrase*.

c* *Significantly different (p = 0.01) from liquid medium at pH 4.8 without ethanol*.

### Sequence analysis

Protein and DNA sequence were recovered from the complete genome sequence of *O. oeni* AWRI_B429 (ACSE00000000) and IOEB-S277 (AZKD00000000). Putative promoter elements were identified by comparison with promoters previously described for *O. oeni* (Labarre et al., 1996) and putative rho independent terminator (hairpins) were identified by using the RNA draw software. Target gene alignment was performed with BioEdit software (7.2.1.) and primers were designed manually and checked by using oligocalculator (http://biotools.nubic.northwestern.edu).

### Growth conditions

The strains were grown in either Grape Juice medium or Semi Defined medium (SMD). The Grape Juice medium contained: commercial red grape juice, 250 g/l, yeast extract, 5 g/l and Tween 80, 1 ml/l. The Grape Juice medium could be supplemented or not with sucrose (10 g/l) and, for solid medium, agar (20 g/l) was added. The medium was sterilized for 20 min at 121°C, 1 bar.

The composition of the SMD medium was: casamino acids (Difco^TM^), 10 g/l, sodium acetate, 3.4 g/l, KH_2_PO_4_, 1 g/l, MgSO_4_, 7 H_2_O, 0.1 g/l, MnSO4, 4 H_2_O, 0.1 g/l, ammonium citrate, 2.7 g/l, bactotryptone, 5 g/l, D/L-malate, 3 g/l, yeast nitrogen base (Difco ^TM^), 6.7 g/l, adenine, uracil, thymine, guanine, 5 mg/l each, and a carbohydrate (either glucose, 20 g/l or glucose and sucrose, 10 g/l each). The carbohydrate solutions were prepared as 10× solutions and were sterilized 20 min at 121°C, 1 bar, while the base was prepared as a 2× solution and sterilized by filtration (0.2 μm cut off).

Before sterilization, the pH of all media was adjusted with H_3_PO_4_ 1M, to 4.8 or less if otherwise indicated in the text.

The red wine used for stress application was a Merlot of 2011 (alcohol 14% v/v, pH 3.6) and the white one, a Sauvignon Blanc of 2010 (alcohol 12.5% v/v, pH 3.3). A Merlot wine of 2012 from appellation Pessac Leognan, Bordeaux, France, was used for survival tests and malolactic rate measurements (pH 3.5, ethanol 12% v/v and malic acid 3.5 g/l, no added sulfites). Laboratory wine was also obtained by fermentation of a Cabernet sauvignon must with *Saccharomyces cerevisiae* X5 (Biolaffort, France). This wine was then modified to produce wines with distinct alcohol content or pH, by ethanol or H_3_PO_4_ addition. The total acidity, sugar, malic acid, SO_2_, pH, and alcohol were analyzed by SARCO laboratory (Floirac, France), using the official methods or those recommended by the International Organization of Viticulture and Wine (OIV).

### Cell concentration measurements

The liquid medium was gently stirred before sampling and the absorbance (600 nm) was measured.

### Capsule observation

To visualize the bacterial capsule, 10 μl of cell suspension were deposited on a microscope slide and mixed with 20% nigrosine aqueous solution and let to dry (5 min). Afterwards, 10 μl of 1% crystal violet was added and the slide was examined under an Olympus BX51 microscope (x100, under oil immersion). The capsule appeared as a white halo around the cells.

### Polysaccharides, malic acid, and sugars quantification

In the case of plate growth assays, 5 ml of NaCl (9 g/l) were added and the colonies were teared-off and solubilized with a rake. This suspension or, in the case of liquid cultures, the whole culture medium, was centrifuged (8,000 × g, 5 min, 4°C), and the pellet was removed. Three volumes of Ethanol-HCl 1 N (95-5) were added to the supernatant to precipitate the polysaccharides. The tubes were let to stand for 24 h at 4°C. Then, they were centrifuged (18,000 × g, 5 min, 4°C), and the pellet was washed with ethanol (80% vol), centrifuged again, dried for 20 min at 65°C and dissolved in distilled water. The amount of neutral polysaccharides was determined by the anthrone sulfuric acid method, using glucose as the standard (Ludwig and Goldberg, 1956). For each sample, the polymer precipitation and assays were done in triplicate.

The malic acid degradation was measured every 2–3 days throughout the fermentations, thanks to a commercial enzymatic kit (Boehringer, France). The glucose and sucrose concentrations were determined by anion exchange chromatography, as described by Dimopoulou et al. (2012).

### Stress application to bacterial culture

When the preculture reached mid exponential growth phase (OD_600nm_ = 0.6), the culture broth was distributed into 10 ml tubes and centrifuged (6,000 × g, 10 min); then, the supernatant was removed and replaced by fresh Grape Juice medium displaying or not a specific stress, or by red or white wine at 25°C. For ethanol shocks, pure ethanol (HPLC quality) was added to grape juice medium at a final concentration of 2, 5 or 10% (v/v). For acid shock, the pH of the Grape juice medium was adjusted to 3 using H_3_PO_4_ 1M. For cold shock, the cells were transferred to cold Grape Juice medium (4°C) and then kept at 4°C until the end of the experiment. The stress was applied for 3 or 24 h. The control condition consisted of the bacterial cells harvested at mid exponential growth phase (OD_600nm_ = 0.6) by centrifugation (6,000 × g, 10 min) and then, re-suspended in fresh Grape Juice medium at 25°C. Cell population was measured before and after the incubation in the different media, through serial dilutions and CFU counts after growth on solid Grape Juice medium. Cell viability was measured based on fluorescent labeling and microscopy according to previous protocol (Laforgue and Lonvaud-Funel, 2012).

### RNA extraction

Cells were harvested by centrifugation (6,000 × g, 10 min, 4°C), then the cell pellet was washed twice with PBS buffer (NaCl 137 mM, KCl 2.7 mM, Na_2_HPO_4_ 10 mM, pH 7) and once with NaCl 0.9%. Between the washing steps, the cells were collected by centrifugation (6,000 × g, 20 min, 4°C).The pellet was re-suspended into 1 ml of an extraction buffer specifically designed for environments rich in polyphenols (Grape Juice and Wine), containing Tris-HCl, pH 8.0, EDTA 300 mM, NaCl 2M, CTAB (cetyltrimethylammonium bromide) 2%, PVPP (polyvinylpolypyrrolidone) 2%, spermidine 0.05% and β-mercaptoethanol 1%. The cells were then transferred into a suitable Fast Prep tube (FP120 Instrument) and vortexed (6 × 45 s, 6,500 × g, 4°C) with sterile glass beads (0.1 mm diameter). After centrifugation (10,000 × g, 15 min, 4°C), 500 μl of the supernatant was transferred into an equal volume of chloroform, to dissolve the hydrophobic particles and separate the proteins and nucleic acids. The mixture was vortexed for 10 min and centrifuged (10,000 × g, 15 min, 4°C). The upper aqueous phase was transferred into an equal volume of isopropanol to precipitate the ribonucleic acid (20 min, −20°C). The RNA pellet was recovered by centrifugation (6,000 × g, 15 min, 4°C), washed twice with 75% ethanol (prepared with DEPC-treated water), dried and finally taken-up into 20 μl of DEPC water. Samples were then treated with DNase as indicated by the manufacturer (DNaA-free^TM^, Ambion). The absence of chromosomal DNA was confirmed by checking the absence of amplification of the malolactic enzyme gene, *mleA* (Divol et al., 2003). The quality of RNA and the efficiency of the extraction were checked by agarose gel electrophoresis (1.5%). RNA concentration was calculated from the absorbance measured at 260 nm (SmartSpec^TM^ Plus spectrophotometer, Biorad). The samples were stored at −80°C, for a maximum period of 1 month. The cDNA were then synthesized using the iScript cDNA synthesis kit (Bio-Rad), as recommended by the manufacturer.

### RT-qPCR

The reaction mixture (20 μL) contained 10 μL of IQ SYBR Green Supermix and 5 pmol/ml of each primer. Primers (Supplemental Table 1) were purchased by Eurofins. The PCR program included an initial denaturation step of 3 min at 95°C, followed by 40 repeating cycles of 10 s at 95°C, 30 s at 60°C and 30 s at 72°C. Fluorescence measurements were performed during each period of elongation. After the last cycle, a melting curve was drawn using the iCycler iQ^TM^ (Bio-Rad) to check PCR specificity. Ranges of cDNA of known concentrations were analyzed by qPCR to construct “standard” curves, characterized by a regression coefficient *(R*^2^*)* and the PCR efficiency (e = ^10^ ^(−1/*slope*)−1^). The validity criteria were *R*^2^ ≥ 0, 985 and 0.85 ≤ e ≤ 1. Relative gene expression was calculated using the ΔΔCT method and every PCR was realized in triplicate (Coucheney et al., 2005).

### Freeze dried cells production

The *O. oeni* strains were prepared in a lyophilized form based on an industrial confidential protocol. Briefly, the cells were grown in an optimized medium (0.5 l), centrifuged and then washed. Industrial maltodextrins were added as cryoprotector and the cells were freeze dried at laboratory scale. The freeze dried cultivable population was determined as previously described (Dimopoulou et al., 2016).

### Survival rate and malolactic fermentation in wine

The freeze dried cells were rehydrated in a water-wine solution (50:50) supplemented with bacterial Energizer nutrient (Biolaffort, France) as recommended by the manufacturer, and in the presence of sucrose (5 g/l) or not. After 17 h at 28°C in this acclimation medium, the bacteria were transferred (2 × 10^6^ CFU/ml) into the indicated wine, previously sterilized by filtration (0.2 μm cut-off). After 48 h of incubation at 28°C in the wines, the survival rate was determined by serial dilutions and colony counts as previously described (Dimopoulou et al., 2016). Strain implantation controls were performed in the middle of MLF according to previous work (Dols-Lafargue et al., 2007).

### Sensory analyses and ester quantification by HS-SPME-GC/MS

Distinct merlot wines were selected to perform sensory analysis. At the end of MLF, the selected wines were sulfured (25 mg/l free SO_2_) and kept for 2 months at 10°C for stabilization. The tests were then performed according to International Organization for Standardization. Panelists were required to give a liking score on products using a 10-point hedonic scale for the descriptors of aromatic complexity, fruit intensity, bitterness, astringency and mouthfeel perception. The panel consisted of 18 trained laboratory researchers of ISVV, Bordeaux University.

Red wine samples were then extracted by HS-SPME and analyzed by GC/MS. The method developed and validated by Antalick et al. (2010, 2012) was used to quantify 32 esters.

### Statistical analysis

The experiments were performed in biological and technological duplicate. *T-*test, one-way or multi-way ANOVA were performed to compare strains performances.

## Results

### EPS specific production is modulated by medium pH and composition

Soluble polysaccharide production by 37 different *O. oeni* strains and final absorbance were determined after 15 days of growth in media displaying distinct pH or additives (ethanol or sucrose). The EPS specific production level (EPS produced/absorbance change in the same time) is reported in Table 1. Whatever the strain considered, no significant soluble EPS production was observed on solid medium. In liquid medium at pH 4.8 (reference medium), the specific EPS production level was highly variable among the strains studied (from 2 to 154 mg/l/AU), with a mean value at 54 mg/l/AU. The strains displaying no *eps2* gene cluster formed part of the lowest producers, even at low pH or in the presence of ethanol. For the strains displaying an *eps2* gene cluster, the decrease of medium pH to 4 did not significantly modify the specific production, while a more acidic medium (pH 3.5), ethanol (5%) or sucrose (1%) addition induced a significant increase of the soluble EPS specific production level, even if distinct behaviors were observed among the studied strains (Table 1).

**Figure 1:**
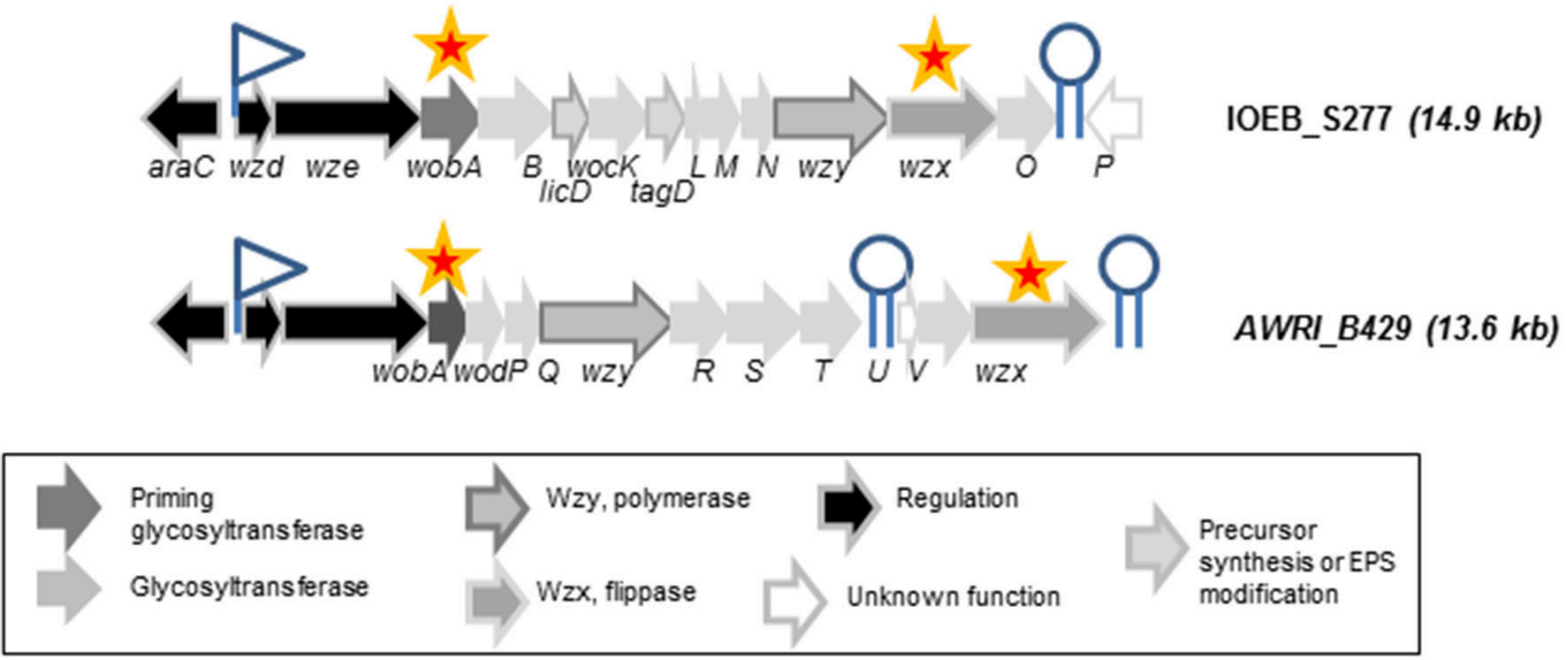
Organization of the *eps2* gene cluster in *O. oeni* IOEB_S277 and AWRI_B429. Putative rho-independent terminators (hairpin structures with polyT) are indicated. A promoter (−10 and −35 consensus sequences) was found upstream *wzd* in both strains. The stars indicate the genes whose expression level was studied by RT-qPCR.

A specific focus was made on three families of strains. Genome survey indicates that two families (the AWRI_B429 and the IOEB_S277) displayed both a complete *eps2* gene cluster (Figure 1) and a *dsrO* gene of 4428 nt encoding a functional dextransucrase according to Dimopoulou et al. (2014). All these strains were encapsulated in all the conditions studied (not shown). In the presence of glucose alone, strains of IOEB_S277 type family produced higher amounts of soluble EPS than strains in family B429 (Table 1). In the presence of glucose and sucrose, half of strains in these two families produced significantly higher amounts of soluble EPS than in the absence of sucrose. The strain VF belongs to a third family of strains: it has a *dsrO* gene of 4428 nt encoding a functional dextransucrase but no *eps2* gene cluster. This strain is never encapsulated and produces no soluble EPS in whatever the growth medium studied (Table 1).

### EPS production and *eps* gene expression kinetics

The strains AWRI_B429 and IOEB_S277 were chosen as model strains. Their *eps2* gene clusters were examined for the presence of putative promoters and terminators (Figure 1). First, the presence of a divergently transcribed regulator gene, *araC*, and a putative promoter suggests a transcriptional regulation level for capsular EPS synthesis. The high similarity of the 5' end of the clusters in the two strains suggests that similar expression pattern may be observed for the 5'end genes in *eps2* cluster. However, two distinct putative terminators were found for strain AWRI_B429 and only one for strain IOEB_S277. This suggests that, in the first strain, genes in *eps2* may not be all co-transcribed. No clear Pribnow box or regulation sequence could be identified next to *dsrO* gene.

**Figure 2:**
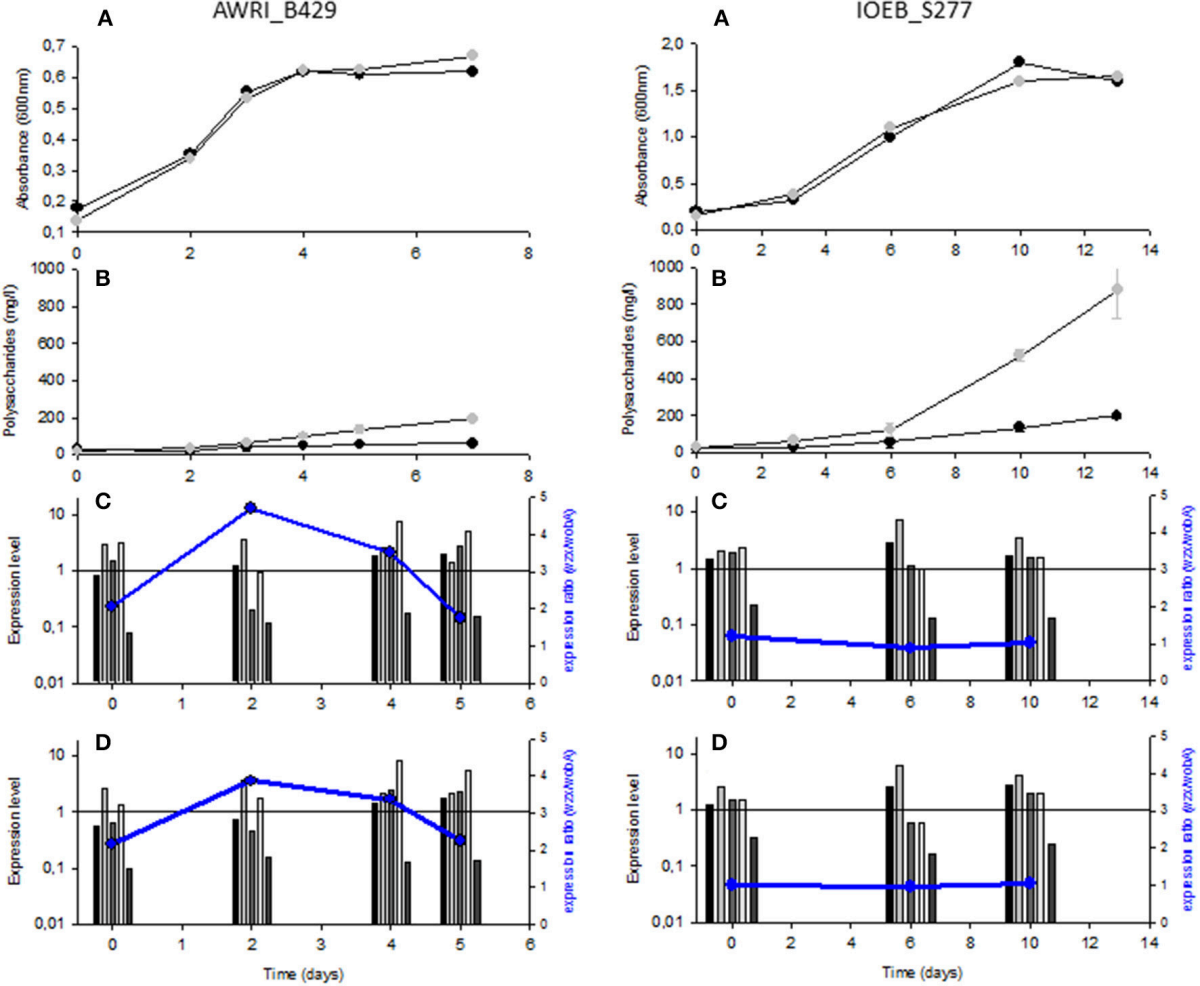
Bacterial growth, EPS synthesis and relative gene expression in SMD medium supplemented or not with sucrose for strains *O. oeni* AWRI*_*B429 and IOEB_S277. **(A)** Bacterial growth on glucose medium (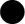) and on glucose+sucrose medium (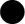). **(B)** Polysaccharide concentration (*n* = 3) on glucose medium (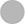) and on glucose+sucrose medium (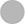). **(C,D)**, Expression level relative to gyrA (2^ΔCT^) at the indicated time: the genes are described in the following order: *pgm, ldh, wobA, wzx* and *dsrO*. The blue point represents the expression ratio between *wzx* and *wobA:* relative gene expression on glucose medium **(C)** and on glucose+sucrose medium **(D)**.

Bacterial growth and exopolysaccharide production were studied in two different growth media (Grape Juice and SMD), supplemented or not with sucrose. Sucrose alone does not support growth of most *O. oeni* strains (Cibrario et al., 2016), but some strains, as the two studied here, can use it to produce dextran (Dimopoulou et al., 2014). The experiment was followed until the stationary growth phase. As a result, the experiment duration varied from one strain or one culture medium to the other. However, for a given strain, similar growth profiles were obtained in the presence as in the absence of sucrose in SMD (Figure 2A). The same was observed in Grape Juice medium (Supplemental figure 1). Very low sucrose consumption (<1 g/l) was observed in both media, while more than 90% of the glucose present was consumed (not shown).

With strain AWRI_B429, in the absence of sucrose, no significant change in EPS concentration was observed, in SMD as in Grape Juice medium (Figure 2B and Supplemental figure 2). On the contrary, in the presence of sucrose, a significant increase of EPS concentration was observed. The EPS maximal concentration reached 200–350 mg/l, when sucrose was present. The strain IOEB_S277 produced EPS during growth in the presence of glucose alone (170 mg/l) and more than 900 mg/l in the presence of glucose + sucrose (Figure 2B).

A capsule was present around the two bacterial strains in all studied media and at all growth stages examined (not shown). However, the method used did not enable to measure the capsule thickness and to visualize if a growth phase was more favorable for capsule formation.

The relative expression level of some *eps* genes was thus studied: two genes of the *eps2* gene cluster, i.e., the priming glycosyltransferase (*wobA*) and the flippase (*wzx)* genes as well as the dextransucrase gene (*dsrO*). The phosphoglucomutase (*pgm*) and the lactate deshydrogenase (*ldh)* genes were studied as controls of the central metabolism (Figures 1A, 2C). By producing Glucose-1-P, the phosphoglucomutase makes the link between catabolism and anabolism and is as key element for heteropolysaccharide synthesis, while lactate deshydrogenase rather reflects the general catabolic activity of the cell. The reference gene used was *gyrA*, as comparison of mean Critical Threshold values (CT) indicated that *gyrA* displayed the highest stability among 4 genes proposed (Desroche et al., 2005; Costantini et al., 2011; Sumby et al., 2012; Peng et al., 2018), for transcription studies in *O. oeni: ldhD, gapA, gyrA*, and *pta* (not shown).

Quite similar gene expression profiles were obtained in the absence as in the presence of sucrose (Figures 2C,D). The *ldh* gene was slightly more expressed during active growth, while the *pgm* gene showed a slightly higher expression during the stationary growth phase. The *eps* genes in the cluster *eps2* were expressed at a level in the same order of magnitude as *gyrA* (relative expression levels between 0.4 and 10). Furthermore, the genes *wobA* and *wzx* were co-expressed in the case of strain IOEB_S277 and slightly less expressed during the active growth phase than during the stationary phase. In the case of strain AWRI_B429 the genes were not co-expressed: *wobA* showed the same profile as with strain IOEB_S277 but *wzx* was always more expressed and the expression ratio *wzx/wobA* varied from 2 to 5 depending on the growth phase or the medium considered, suggesting the existence of a second promoter in the *eps2* gene cluster, between *wobA* and wzx.

The gene *dsrO* was significantly less transcribed than the other *eps* genes and its expression level was almost not modified along growth in liquid media. Furthermore, its expression level did not change in the presence of sucrose (Figures 2C,D). According to gene expression, the *O. oeni* cells would therefore be more ready to synthesize capsular EPS at the end of the growth phase (increased expression of the genes of the *eps2* cluster and of the *pgm*) and would be always moderately ready to produce dextran (constant expression of *dsrO*).

### Evolution of *eps* gene expression in response to medium change

**Figure 3:**
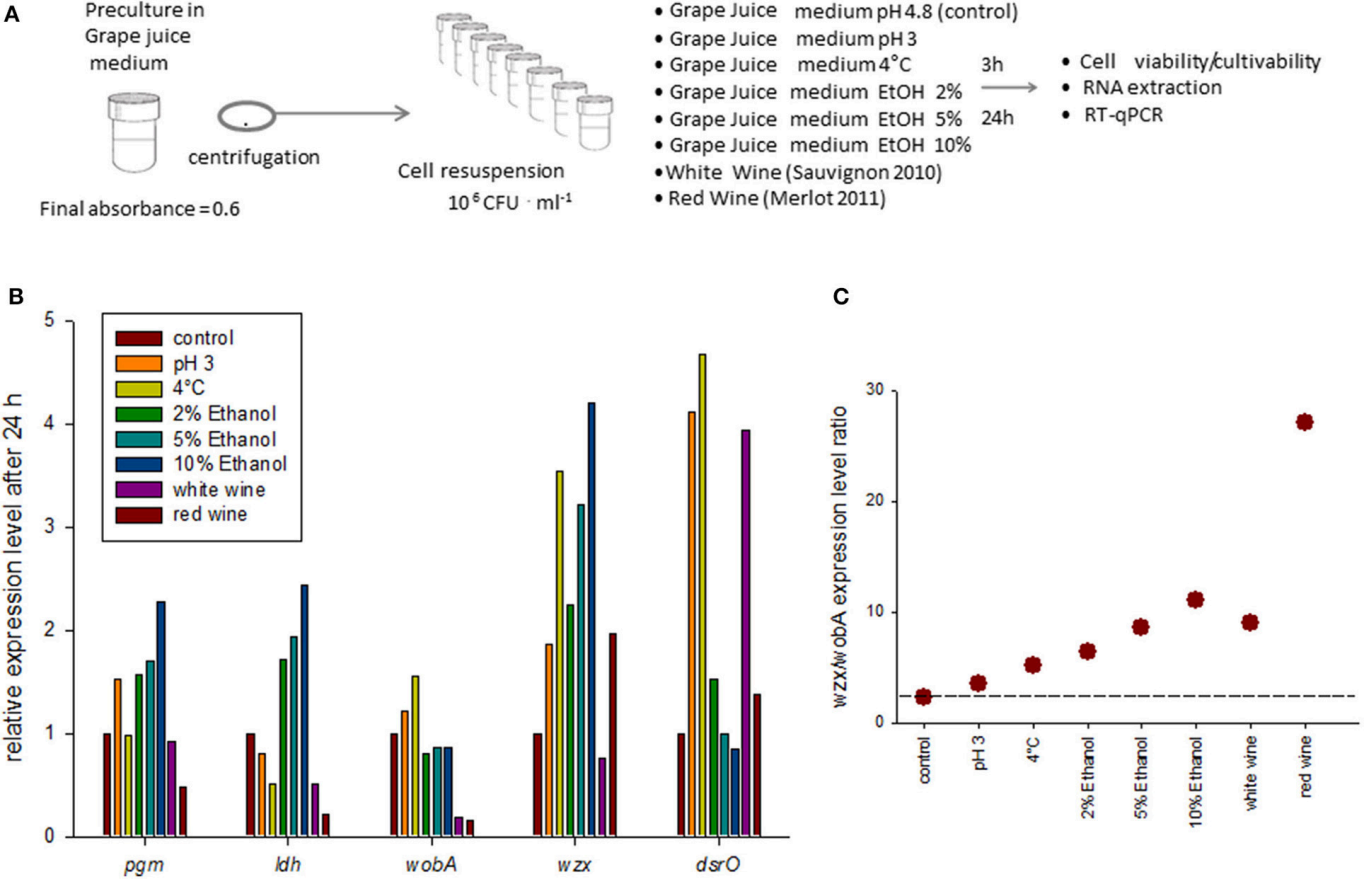
Expression of *eps* genes in response to medium change in *O. oeni* AWRI_B429.**(A)** Method: the bacterial cells were initially grown in Grape Juice medium until absorbance reached 0.6. After centrifugation, they were re-suspended in 8 different fresh growth media. After 3 and 24 h of incubation in the new medium, the cell cultivability (CFU counts) and the cell viability (fluorescent labeling and microscopy) were measured, and RNA extraction and RT-qPCR were realized. **(B)** Relative gene expression level of target genes after 24 h of incubation in different culture media. The reference gene was *gyrA*. The target gene expression level relative to *gyr A* (2^ΔCT^) was then compared to the relative expression in the control condition (2^ΔΔCT^). The relative expression in control condition was set to 1 for each gene. The results after 3 h of incubation are shown in Supplemental figure 2. **(C)** Expression level ratio of *wzx*/*wobA* after 24 h (see Supplemental figure 2 for expression level after 3 h).

We then examined whether the *eps* gene expression was modulated by single stresses relevant to wine or during winemaking: acidity, low temperature and different alcohol concentrations (2, 5, and 10%), or during direct inoculation into white and red wine (Figure 3A). In most cases, the cell cultivability decreased after medium change. It was comprised between 50 and 100% of that observed in the control condition (not shown) for all media studied. Indeed, red wine was the most harmful (10% remaining cultivability) and viability tests (epifluorescence analysis) indicated that only 40% of the cells were viable after 24 h of incubation in red wine, suggesting the presence of a high proportion of VBNC cells in this specific case.

The application of stress and cell mortality may destabilize the expression of the reference gene. Among *ldhD, gapA, gyrA*, and *pta*, the gene showing the lowest dispersion in terms of standard deviation was *gyrA* and it was used as an internal calibration gene for this part of the study also.

Three hours after medium change, the studied gene expression levels were weakly modified (factor 2 maximum). The expression of *wzx* in the presence of 10% of ethanol was the only which really stood out (x3 compared with the control condition, Supplemental figure 2). Overall, cold shock and acidic pH decreased the expression of genes associated with central metabolism (*ldh* and *pgm*), as those associated with EPS synthesis (*wobA, wzx* and *dsrO*). Ethanol, on the other hand, slightly stimulated the expression of some of the studied genes, in a dose-dependent manner. The wines slightly stimulated the expression of *pgm* and *dsrO*, but decreased that of genes in *eps2* cluster, and had little effect on *ldh* expression.

The changes in expression level were more significant after 24 h (Figure 3) which suggests that these genes are associated with adaptation to medium change more than to resistance to stress The low pH and temperature as well as the increasing alcohol concentrations clearly stimulated the expression of *wzx* and *dsrO* and moderately that of the central genes *pgm* and *ldh*. The gene *wobA* was almost unaffected. These modifications are not observed in tested wines except for the case of *dsrO* in white wine. The expression ratio *wzx*/*wobA*, increased whatever the medium change and the increase was particularly high in red wine (Figure 2).

### Use of media inducing *eps* genes or EPS production to improve malolactic starter survival during freeze drying

Previous work has shown that strains producing dextran in the presence of sucrose added in industrial growth media were more tolerant to freeze drying (Dimopoulou et al., 2016). We examined if the application of a stress to stimulate the *eps* expression and EPS specific production level during malolactic starter growth, could increase strain survival during the subsequent freeze drying process. We focused on the two model strains (AWRI_B429 and IOEB_S277). The tests were made in glucose + fructose as in glucose + fructose + sucrose containing growth media. Ethanol (5%) addition in the medium or a cold incubation (4°C), for 3 or 24 h just before freeze drying, didn't give the expected results (not shown). More acidic growth media were not assayed as they always led to much lower final biomass yields.

### Use of media inducing *eps* genes and EPS production to improve malolactic starter survival and performance during inoculation in wine

**Figure 4:**
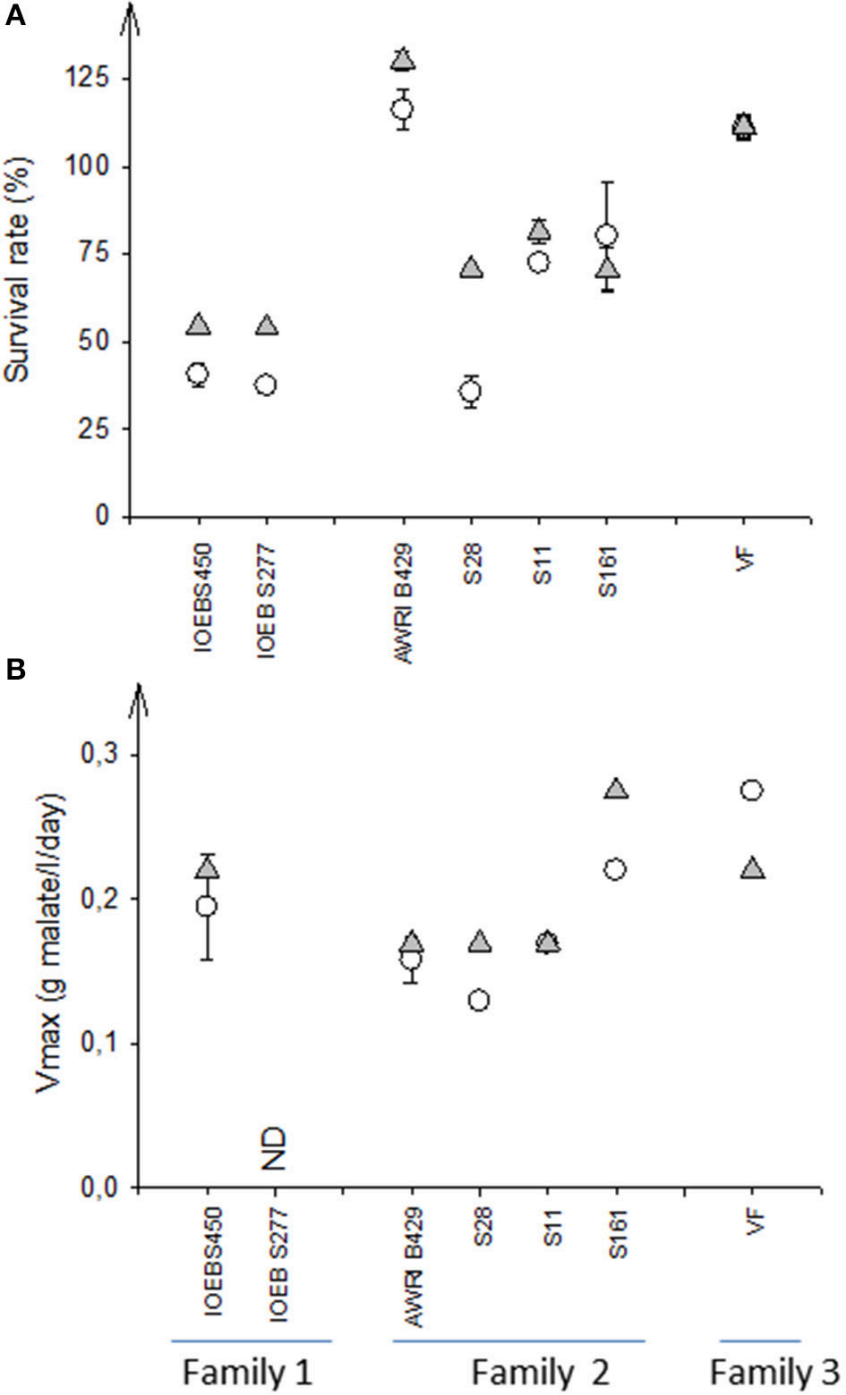
Influence of the acclimation medium (standard or supplemented with sucrose) on survival **(A)** and malolactic fermentation kinetics **(B)**. Seven *O. oeni* strains, belonging to 3 distinct families regarding their *eps* genes, were produced at laboratory scale in a freeze dried form, then acclimated and inoculated in wine (Pessac Leognan, 2012). The survival rate was calculated by comparing the initial cell cultivability (inoculum) with the cultivability after 48 h in wine; the Vmax of the malolactic fermentation was the ratio of the initial concentration of malic acid to the days needed to complete its consumption. Error bars represent the standard deviation of two biological replicates. 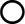 acclimation to wine performed in standard conditions. 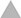 acclimation to wine performed in the presence of 5 g/l sucrose.

The only way to reproducibly enhance bacteria survival to freeze drying thanks to EPS production was the addition of sucrose to the growth medium in order to activate the dextransucrase pathway (Dimopoulou et al., 2016). We therefore examined if the presence of sucrose could also improve the bacterial survival during inoculation into the wine. Seven strains of *O. oeni* belonging to the three different families described (VF non EPS producer, AWRI_B429 and IOEB_S277 EPS producers with different *eps2* cluster) were first produced and freeze-dried. Then, they were acclimated during 17 h into diluted wine supplemented or not with sucrose (5 g/l). EPS formation during the 17 h of acclimation was undetectable (too high background signal). However 1 week incubation in the same conditions led to significant production of EPS, only when sucrose was present, in the case of strains in AWRI_B429 and IOEB_S277 families. After the 17 h of acclimation, the strains were inoculated into a Merlot red wine. The survival rate and the fermentation kinetics were followed (Figure 4). Our results showed a high level of dispersion of the strain performances, even inside a given family. The origin of the strain may also direct the fermentation capacity (Campbell-Sills et al., 2017). Nevertheless, the presence of sucrose in the acclimation medium generally displayed a positive effect on both the survival and the fermentation rate. The use of sucrose negatively alters the MLF rate only in the case of VF, which does not produce EPS.

This experiment was repeated with the strains S28, S11 and IOEB_S450 in a laboratory Cabernet Sauvignon red wine modified to mimic “difficult wines” (pH 3.2, ethanol 14 and 16% vol). Whatever the characteristics of the tested laboratory wine, the use of sucrose in the acclimation medium improved the survival rate of strain S28 and had slightly positive but non-significant effect on that of the two other strains (not shown).

**Figure 5:**
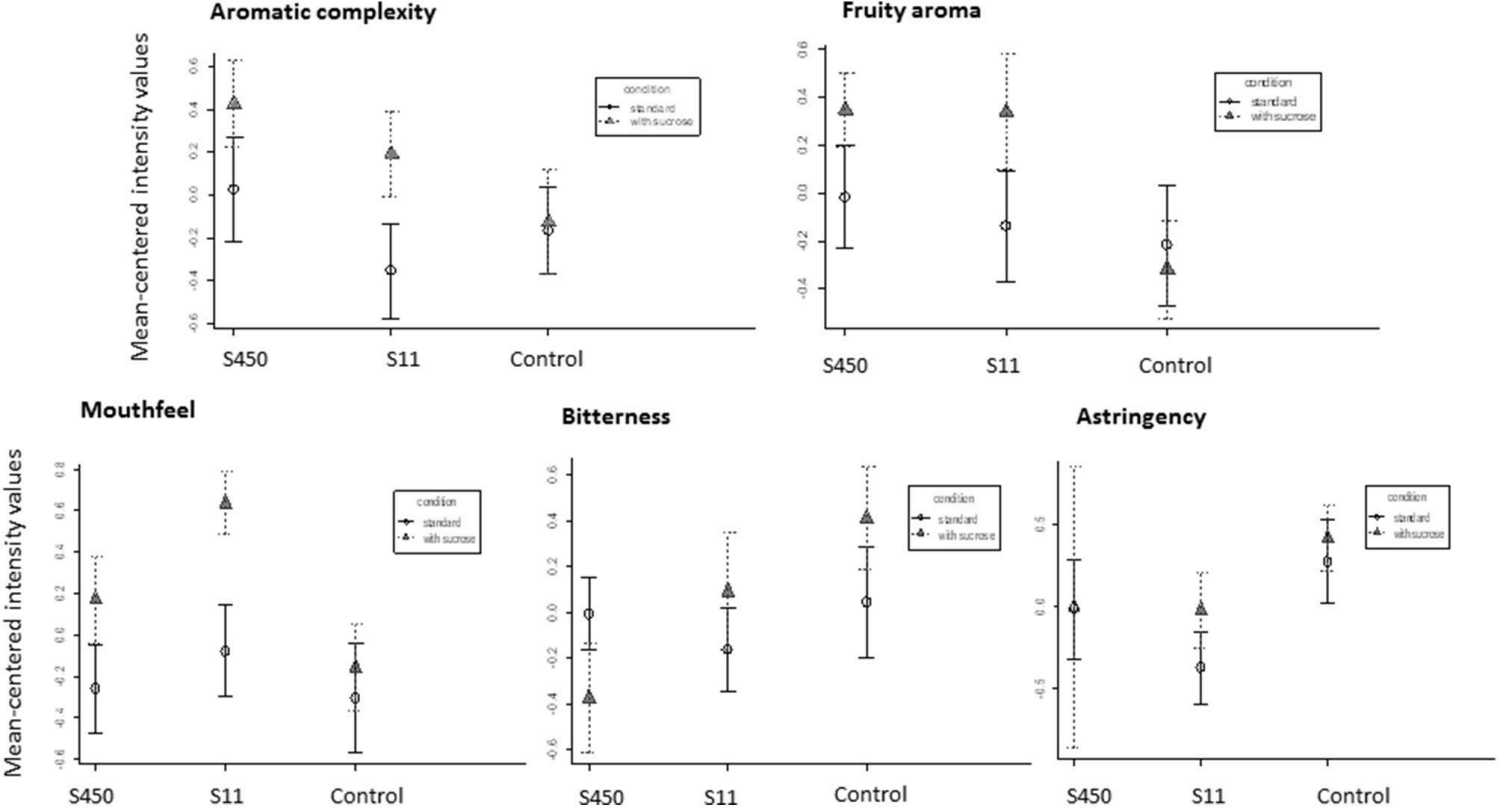
Mean-centered intensities of sensory characteristics descriptors of wines inoculated with two different *O. oeni* strains (IOEB_S450 and S11) after acclimation without or with sucrose. The control is the wine that didn't realize the malolactic fermentation in which sucrose was added (5 mg/l) or not to mimic sucrose addition via the acclimated inoculum. The wine used for the experiment was a Merlot from 2012 (pH 3.5, ethanol 12% v/v and malic acid 3.5 g/l, no added sulfites). Error bars represent the standard deviation of two biological replicates.

The produced Merlot wines were finally tasted in order to verify that the addition of sucrose in the acclimation medium didn't affect the sensory quality of the final product (Figure 5). On the contrary, when the strains S11 and IOEB_S450 (belonging to AWRI_B429 and IOEB_S277 families respectively), were acclimated in the presence of sucrose (5 g/l), the wines after MLF were better evaluated, when compared to those produced with the control acclimation medium (no added sucrose). The descriptors examined were the aromatic complexity, the fruity aroma intensity, the mouthfullness, bitterness and astringency. The fruity aroma was improved for both strains when acclimated with sucrose, while the mouthfeel sensation and the aromatic complexity were significantly increased for strain S11.

The same wines were quantified for thirty two esters recognized as key aromatic compounds of MLF (Antalick et al., 2012). The total ester amount was similar for the two strains in both tested conditions, which confirmed their specific production during MLF (Supplemental figure 3).

## Discussion

Much effort has been made to improve malolactic starter performance, from genetic modification to directed evolution. However these methods could be time consuming, not always efficient or rejected by the consumer (Dicks, 1994; Assad-García et al., 2008; Betteridge et al., 2015; Darsonval et al., 2016; Jiang et al., 2018). The use of endogenous EPS recently appeared as an interesting alternative (Dimopoulou et al., 2016). EPS are multifunctional tools contributing to the survival and subsequent colonization of various ecosystems by non-pathogenic but also pathogenic bacteria (Cerning, 1990; Looijesteijn et al., 2001; Mah and O'Toole, 2001; Weiser et al., 2001; Lu and Rock, 2006; Dols-Lafargue et al., 2008; Coulon et al., 2012). Previous work has shown that all *O. oeni* strains displayed *eps* genes and that most of them produce capsular but also soluble EPS (Dimopoulou et al., 2012, 2014) that could be useful for producing freeze dried strains or during cellar colonization (Bastard et al., 2016; Dimopoulou et al., 2016).

In this paper, we show that the *eps* genes are expressed at a significant level (equal to the level of expression of the reference gene) all along growth in the tested media supplemented with or not with sucrose. The need for EPS formation may thus be permanent in the conditions studied. However, the low soluble EPS concentrations observed and the inability to measure the thickness of the EPS capsule make it difficult to determine if EPS are produced more actively during a specific growth phase. Furthermore, many single constraints relevant in wine modify the corresponding mRNA concentrations particularly after 24 h. The absence of significant change in certain glycosyltransferase genes expression after short period of time of stress application is in accordance with previous transcriptomic studies (Olguín et al., 2015; Bastard et al., 2016; Margalef-Català et al., 2016; Liu et al., 2017). Moreover, their increased expression after 24 h suggests that the *eps* genes are not really involved in the immediate response to stress, but rather in adaptation to new environmental conditions (Guzzo et al., 2000; Bourdineaud et al., 2003; Da Silveira et al., 2003; Maitre et al., 2014; Dimopoulou et al., 2017). Consequently, the EPS liberation is stimulated in acidic or alcohol containing media and we observed increased specific production rates after 2 weeks of incubation. Stressful conditions, which slow bacterial growth (and consequently cell wall synthesis) and increase *eps* and *pgm* gene expression, may allow a better availability of the isoprenoid lipid carrier and the nucleotide sugars precursors. This may lead to increased polysaccharide production by an increased pool of glucosyltransferase (Levander et al., 2001). However, the inability to quantify the EPS capsule makes it difficult to determine if the proportion between soluble and capsular EPS is modified in specific conditions. On the contrary, the growth on solid medium does not stimulate soluble EPS formation by *O. oeni*, suggesting that these polymers are not needed for the consolidation of the colonies, unless very adhesive molecules are formed in such conditions.

In *O. oeni* AWRI_B429, the presence of multiple potential promoters and terminators in the same cluster is in contrast to what described for most *eps* operons in lactic acid bacteria (Stingele et al., 1996; van Kranenburg et al., 1997; Jolly and Stingele, 2001; Lamothe et al., 2002), even if exceptions have been mentioned (Péant et al., 2005). The relative expression levels of the various *eps* gene confirmed the independent transcription of the priming glycosyltransferase and the flippase genes. This may modify the EPS biosynthetic pathway limiting step and may induce difference in EPS structure or size, in response to medium change. Experiments may be carried out in the future to examine this specific point. In this specific strain, its seems that, after medium change, the cells need more EPS but they may also need different EPS or even both, as shown for cyanobacteria (Ozturk and Aslim, 2010).

The dextransucrase gene *dsrO* is not induced by sucrose, contrarily to what described in *Leuconostoc mesenteroides* or other glycoside hydrolase in lactic bacteria (Neely and Nott, 1962; Quirasco et al., 1999; Schwab and Gänzle, 2006) and *O. oeni* behave similarly *Lactobacillus sakei* or *Weisella confusa* (Bounaix et al., 2010; Nácher-Vázquez et al., 2017), or even *Leuconostoc mesenteroides* NRRL_1299 for minor dextransucrases (Dols et al., 1998). Taking into consideration the unusual presence of sucrose in the usual environment of *O. oeni*, the constitutive expression of *dsrO* is quite surprising and suggests that *O. oeni* must always be prepared to produce dextran and that dextran is very useful for the bacteria. The previously demonstrated protective role of the produced dextrans during freeze drying (Dimopoulou et al., 2016) is in favor of this hypothesis. The constitutive expression of *dsrO* also constitute a selective advantage during the acclimation step: sucrose addition into the acclimation medium increased most freeze-dried strains survival to inoculation in wine, and enabled faster MLF, even though the dextran concentration produced is very low. Several bacterial EPS were shown to be involved in bacterial protection (Ionescu and Belkin, 2009; Ruas-Madiedo et al., 2009; Coulon et al., 2012; Varin et al., 2012) but the protective role is more rarely coupled with fermentation rate improvement.

Interestingly the acclimation in the presence of sucrose also improved the sensory attributes of wine. Since specific wine polysaccharides, especially mannoproteins, have been proven to modify wine astringency and sucrosity (Taira et al., 1997; Moine, 2009), the possible effects of dextran were examined: noticeable effects were observed with low MW dextrans (1 kDa) at concentrations higher than 1 g/L (Moine and Iturmendi, unpublished results). Since the dextran concentration formed during acclimation in the presence of sucrose and then introduced into the wine is at most 2.5 mg/L (taking into account the inoculation rate of 1%), its seems unlikely that the sensory effects observed are linked to dextran itself and controls show that they are no more linked to the remaining sucrose. Since the aromatic profile was improved for both tested strains, we can assume that the sucrose addition may also activate enzymatic pathways resulting in ameliorated aromatic profile.

## Conclusion

The fact that all the *O. oeni* strains are equipped with at least one EPS biosynthetic pathway and the *eps* genes are expressed at significant levels whatever the growth conditions, points out the importance of these polymers for the species. These polymers may form part of the tools that render *O. oeni* able to grow in various niches like grapes, must and wine. The gene *dsrO* appears as a promising simple and original tool, because dextran production pathway is less energy demanding than the other EPS biosynthetic pathway (Hanson et al., 2011) and because it offers increased bacterial protection thus leading to ameliorated fermentation rates and better wine quality.

## Author contributions

MD, VM, JC, and MD-L designed the experiments. MD, JR, OC, CM-S, and NI made the experiments. MD and MD-L prepared the manuscript. All authors read and approved the final manuscript.

### Conflict of interest statement

The authors declare that the research was conducted in the absence of any commercial or financial relationships that could be construed as a potential conflict of interest.
