# Supplementary figures and images for: Oenococcus oeni Exopolysaccharide Biosynthesis, a Tool to Improve Malolactic Starter Performance
Source: Front Microbiol. 2018 Jun 12;9:1276. doi: 10.3389/fmicb.2018.01276 (PMC6006919; doi:10.3389/fmicb.2018.01276)

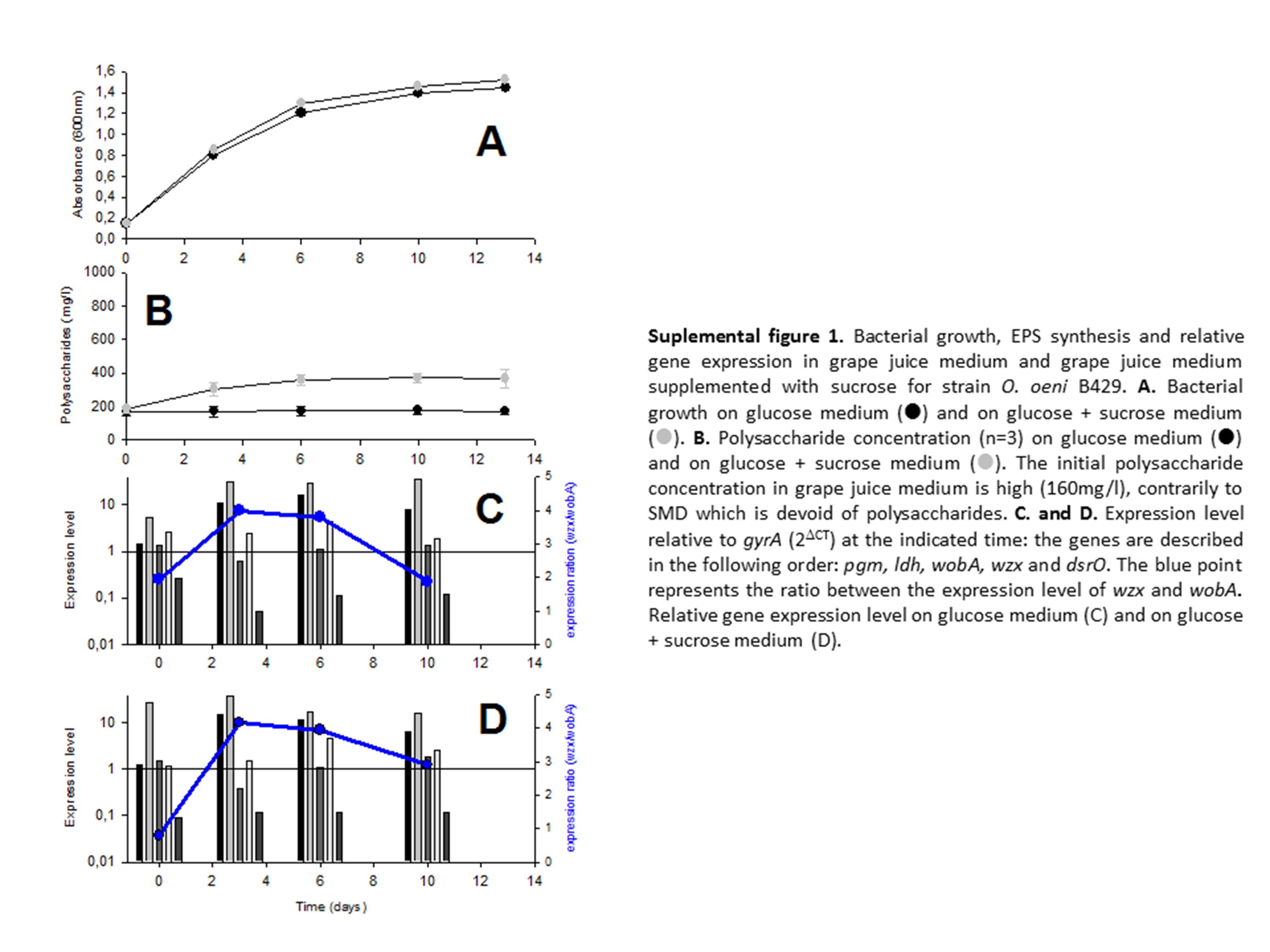

Supplement: Supplementary file 1 [file Image_1.TIF]

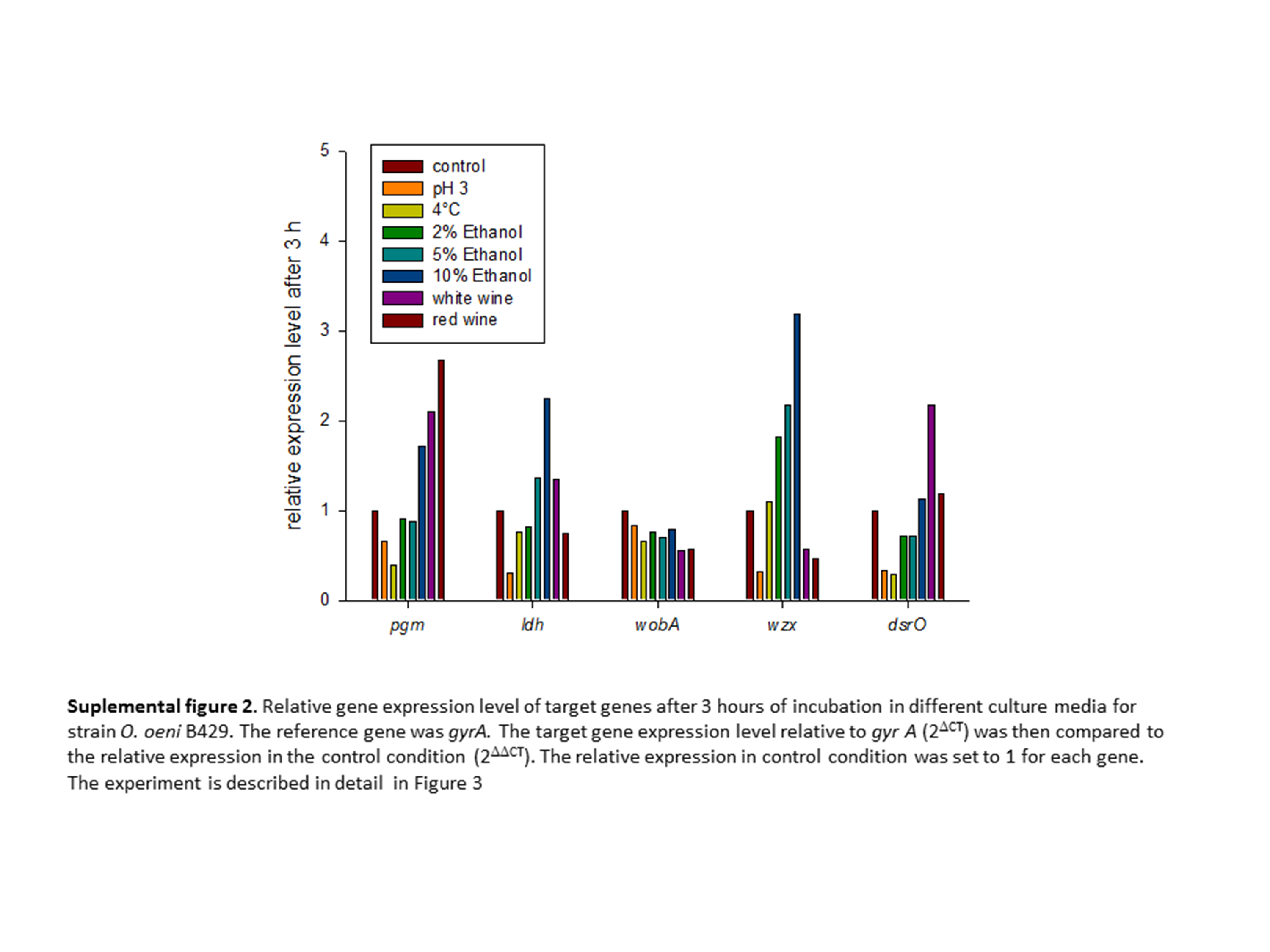

Supplement: Supplementary file 2 [file Image_2.TIF]

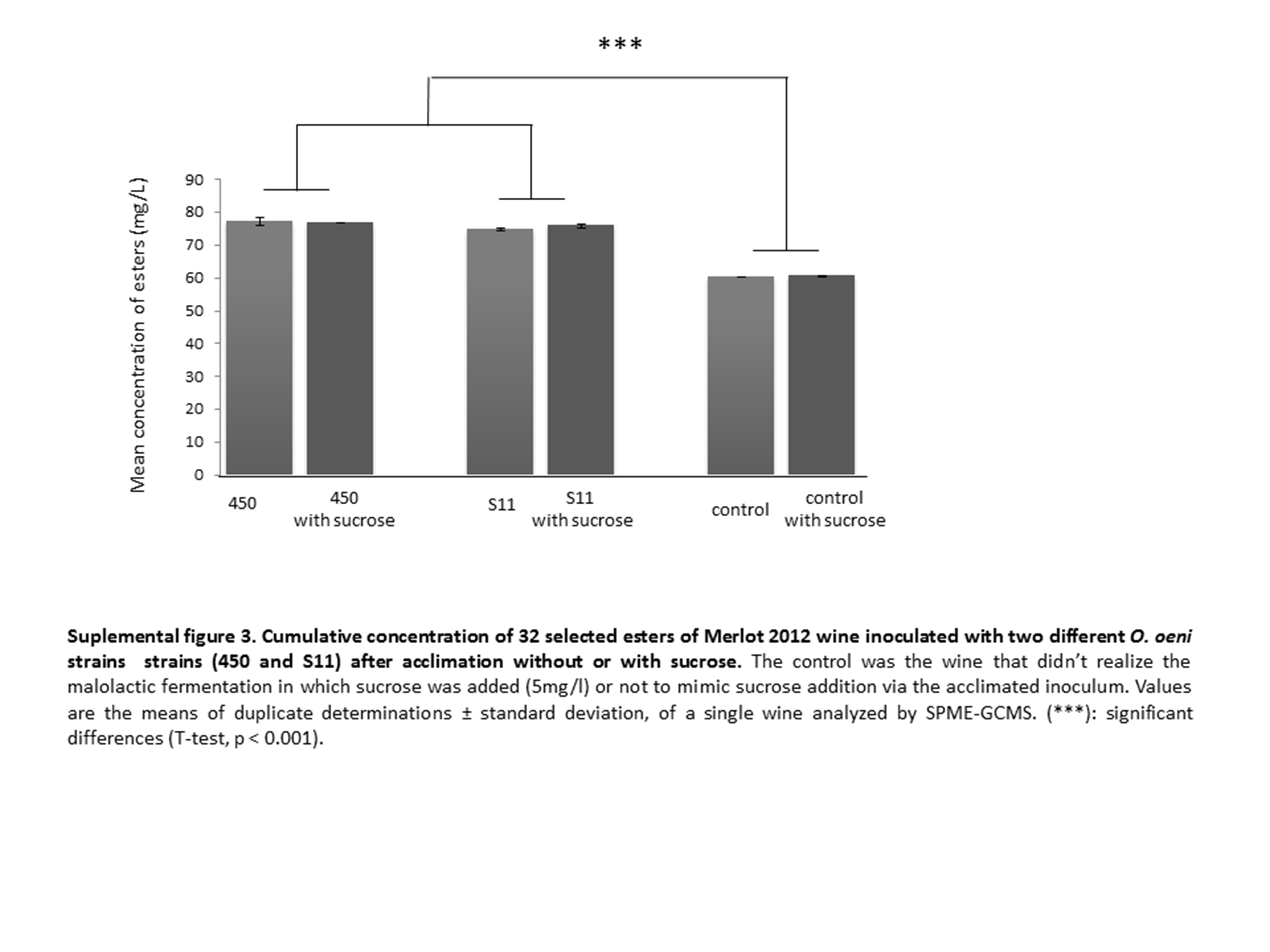

Supplement: Supplementary file 3 [file Image_3.TIF]
