# Supplementary material for: Oenococcus oeni Exopolysaccharide Biosynthesis, a Tool to Improve Malolactic Starter Performance
Source: Front Microbiol. 2018 Jun 12;9:1276. doi: 10.3389/fmicb.2018.01276 (PMC6006919; doi:10.3389/fmicb.2018.01276)
Supplement: Supplementary file 4 [file Table_1.docx]

**Supplemental TABLE S1** List of studied gene and corresponding primers sequences

| Gene | Encoded protein function | Primers | Reference |
| --- | --- | --- | --- |
| *ldh* | D-lactate deshydrogenase | GCCGCAGTAAAGAACTTGATG  TGCCGACAACACCAACTGTTT | (1) |
| *gyrA* | DNA gyrase subunit A | CGCCCGACAAACCGCATA  CAAGGACTCATAGATTGCCG | (1) |
| *ptA* | Phosphotransacetylase | CGGCAGCTATGTGGCTGCAGGA  GCGGGACGAACCGTATCGCC | (2) |
| *gapA* | D-glyceraldehyde-3-P-deshydrohenase | AGACGCTCGTAACCTTCCTTGGGTTA  GGCAGGTGCAGAAATAATACCCGC | (2) |
| *pgm* | phosphoglucomutase | CCGCATCCGACTCCGGAAGAG  GATTTCATCGGCAGGTTTGGCGGC | (3) |
| *dsrO* | dextransucrase | CACAGTGGCTAAAAGAGGTCATCACGAAT  ggtgtccgattcaacaagcgatagtc | This study |
| *wobA* | Priming glycosyltransferase, cluster *eps2* | CGGGTTTGCATTTTATCCGGGTAC  GGGAAGCTATGGCAAATCCTACCAG | This study |
| *wzx* | Flippase, cluster *eps2* | CTCGATTCTCAATTCAAGCGTTGCTAG  CAAATTACCTAAGCTCGCTGTGACTGCAC | This study |

1. **Desroche N, Beltramo C, Guzzo J**. 2005. Determination of an internal control to apply reverse transcription quantitative PCR to study stress response in the lactic acid bacterium *Oenococcus oeni*. J Microbiol Methods **60**:325–333.

2. **Sumby KM, Grbin PR, Jiranek V**. 2012. Validation of the use of multiple internal control genes, and the application of real-time quantitative PCR, to study esterase gene expression in *Oenococcus oe*ni. Appl Microbiol Biotechnol **96**:1039–1047.

3. **Costantini A, Vaudano E, Rantsiou K, Cocolin L, Garcia-Moruno E**. 2011. Quantitative expression analysis of *mleP* gene and two genes involved in the ABC transport system in *Oenococcus oeni* during rehydration. Appl Microbiol Biotechnol **91**:1601–1609.
